# Transcriptome Analysis of *Paralichthys olivaceus* Erythrocytes Reveals Profound Immune Responses Induced by *Edwardsiella tarda* Infection

**DOI:** 10.3390/ijms21093094

**Published:** 2020-04-28

**Authors:** Bin Sun, Xuepeng Li, Xianhui Ning, Li Sun

**Affiliations:** 1 CAS Key Laboratory of Experimental Marine Biology, CAS Center for Ocean Mega-Science, Institute of Oceanology, Chinese Academy of Sciences, 7 Nanhai Road, Qingdao 266071, China; sunbin17@mails.ucas.ac.cn (B.S.); lixuepeng14@mails.ucas.ac.cn (X.L.); xhningouc@163.com (X.N.); 2 Laboratory for Marine Biology and Biotechnology, Qingdao National Laboratory for Marine Science and Technology, 1 Wenhai Road, Qingdao 266237, China; 3 University of Chinese Academy of Sciences, 19 Yuquan Road, Beijing 100049, China

**Keywords:** *Paralichthys olivaceus*, red blood cells, *Edwardsiella tarda*, transcriptome, immune response

## Abstract

Unlike mammalian red blood cells (RBCs), fish RBCs are nucleated and thus capable of gene expression. Japanese flounder (*Paralichthys olivaceus*) is a species of marine fish with important economic values. Flounder are susceptible to *Edwardsiella tarda*, a severe bacterial pathogen that is able to infect and survive in flounder phagocytes. However, the infectivity of and the immune response induced by *E. tarda* in flounder RBCs are unclear. In the present research, we found that *E. tarda* was able to invade and replicate inside flounder RBCs in both in vitro and in vivo infections. To investigate the immune response induced by *E. tarda* in RBCs, transcriptome analysis of the spleen RBCs of flounder challenged with *E. tarda* was performed. Six sequencing libraries were constructed, and an average of 43 million clean reads per library were obtained, with 85% of the reads being successfully mapped to the genome of flounder. A total of 1720 differentially expressed genes (DEGs) were identified in *E. tarda*-infected fish. The DEGs were significantly enriched in diverse Gene Ontology (GO) terms and Kyoto Encyclopedia of Genes and Genomes (KEGG) pathways, especially those associated with immunity, disease, and infection. Ninety-one key DEGs involved in 12 immune-related pathways were found to form extensive interaction networks. Twenty-one genes that constituted the hub of the networks were further identified, which were highly regulated by *E. tarda* and involved in a number of immune processes, notably pathogen recognition and signal transduction, antigen processing and presentation, inflammation, and splicing. These results provide new insights into the immune role of flounder RBCs during bacterial infection.

## 1. Introduction

Red blood cells (RBCs) are the most abundant cell type in the blood. While the mammalian mature RBCs lack nuclei, the RBCs of non-mammals contain nuclei and cytoplasmic organelles, which suggests the ability to synthesize proteins in response to stimuli [1,2,3,4]. Recently, nucleated RBCs have been reported to be involved in various immune responses when stimulated with pathogens such as *Escherichia coli* [5], *Candida albicans* [6,7], infectious salmon anemia virus (ISAV) [8], piscine orthoreovirus (PRV) [2], non-replicating viruses like viral hemorrhagic septicemia virus (VHSV) [9], and infectious pancreatic necrosis virus (IPNV) [1,10]. The nucleated RBCs express pattern recognition receptors (PRRs) that recognize pathogen-associated molecular patterns (PAMPs) on microorganisms [11,12]. In fish, rainbow trout and Atlantic salmon RBCs express Toll-like receptor (TLR) 3 and TLR9 that recognize CpG motifs [7,13,14,15]. Atlantic salmon RBCs also express RIG-I that interacts with intracellular viral dsRNA [14]. NOD2, NLRX1, *NLRC3*, and NLRC5 receptors of NOD-like receptors (NLRs) family were detected in rainbow trout RBCs stimulated with VHSV [11]. The activation of the PRRs of nucleated RBCs has been reported to trigger signal transductions and induce the transcription of diverse genes related to innate immunity, such as interferon, interleukin, chemokines, and antimicrobial peptide [12]. In addition, nucleated RBCs also display an antigen-presenting cell (APC)-like behavior, and fish RBCs have been reported to express MHC-I and MHC-II [16,17,18,19,20]. The MHC-I and MHC-II exposed on the surface of RBCs can be recognized by T cells and activate the adaptive immunity [12].

*Edwardsiella tarda* is a gram-negative bacteria that is known to infect a wide range of hosts, including birds, reptiles, mammals, and fish [21,22]. It is a severe pathogen to many farmed fish species, including Japanese flounder (*Paralichthys olivaceus*), and has caused huge economic losses in aquaculture [23]. Studies have shown that *E. tarda* exhibits a strong capacity to evade host immune responses and is able to replicate in host macrophages and resist the killing effect of serum complements [24,25]. A recent study revealed that *E. tarda* markedly suppressed the induction of a large amount of immune genes, notably RIG-I-like receptors, cytokines, and interferon-related genes, during its infection of mammalian macrophages [26]. In a previous report, we demonstrated that Japanese flounder RBCs were capable of ingesting both live and inactivated *E. tarda*, the former via clathrin-mediated endocytosis [27]. However, the immune response induced by *E. tarda* in flounder RBCs has not been investigated.

In this study, we examined the capacity of *E. tarda* to invade and replicate in flounder RBCs, and analyzed the transcriptome of flounder spleen erythrocytes induced by *E. tarda* challenge. We identified a large amount of differentially expressed genes (DEGs) and analyzed their functional enrichment in Gene Ontology (GO) and Kyoto Encyclopedia of Genes and Genomes (KEGG) pathways. Further, we constructed protein–protein interaction networks to reveal the key immune-related DEGs involved in *E. tarda* infection. Our results provided a valuable molecular basis for further study of the mechanism of flounder erythrocytes against bacterial infection.

## 2. Results

### 2.1. In Vitro Infection of E. tarda in RBCs

**Figure 1:**
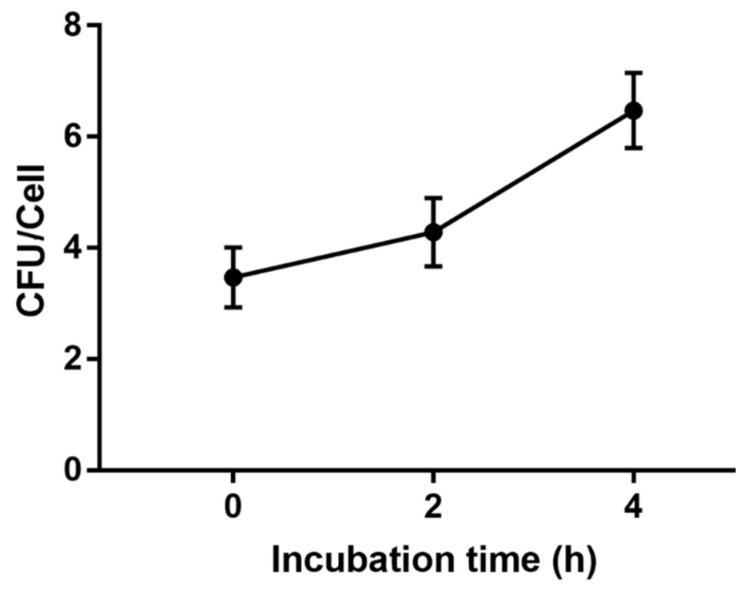
Intracellular replication of *Edwardsiella tarda* in flounder red blood cells (RBCs). RBCs were infected with *E. tarda* for 3 h, and the extracellular bacteria were killed with antibiotic. The cells were then incubated for 0 h, 2 h, or 4 h, and the number of intracellular bacteria (shown as Colony Forming Unit, CFU) was determined. Data are presented as means ± SEM of three independent experiments.

Our previous study indicated that *E. tarda* could invade into flounder RBCs [27]. To examine whether *E. tarda* was able to replicate inside RBCs, the bacteria attached to the surface of RBCs were killed with antibiotics, and the cells were incubated further for 2 h and 4 h. Plate count showed that the intracellular bacterial number increased with the incubation time (Figure 1), indicating an ability of *E. tarda* to replicate inside RBCs.

### 2.2. In Vivo Infection of E. tarda in Flounder Blood and Spleen Erythrocytes

**Figure 2:**
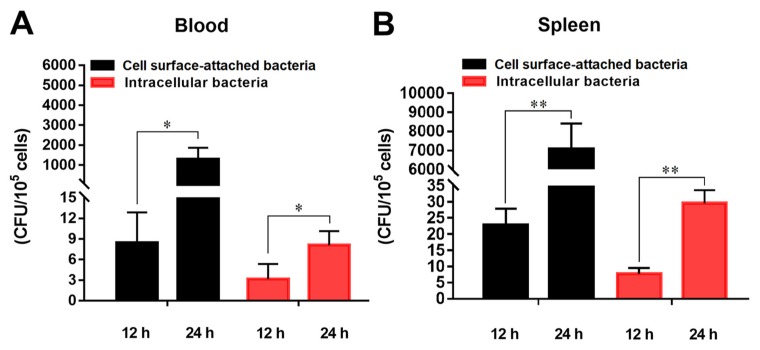
In vivo infection of *Edwardsiella tarda* in flounder blood and spleen erythrocytes. Flounder were infected with or without (control) *E. tarda* for 12 h and 24 h, and erythrocytes were collected from blood (**A**) and spleen (**B**). The cell surface-attached and intracellular *E. tarda* were determined and shown as Colony Forming Unit (CFU). Data are presented as means ± SEM of three independent experiments. *, *p* < 0.05; **, *p* < 0.01.

For in vivo infection, flounder were infected with *E. tarda* for 12 h or 24 h. Erythrocytes were collected from the blood and spleen of the fish and purified to high purity (≥98%) (Figure S1). Both cell surface-attached and intracellular *E. tarda* were detected in the erythrocytes of the infected fish (Figure 2). No *E. tarda* were detected from the erythrocytes of the uninfected control fish. In *E. tarda*-infected fish, both cell surface-attached and intracellular bacterial numbers increased significantly from 12 h to 24 h (Figure 2). Furthermore, at both time points, the numbers of intracellular *E. tarda* in spleen erythrocytes were much higher (2.5 and 3.7 times, respectively) than that in blood erythrocytes, suggesting a more robust bacteria–host cell interaction in spleen RBCs. For this reason, as well as the fact that spleen is one of the major immune organs and an important origin of erythropoiesis in teleost, the spleen erythrocytes from infected and uninfected fish were used for subsequent transcriptome analysis described below.

### 2.3. RNA-seq of the Spleen Erythrocytes from E. tarda-Infected and Uninfected Flounder

To examine *E. tarda*-induced transcription profiles in erythrocytes, three libraries were constructed with the RNAs from the spleen erythrocytes of *E. tarda*-infected flounder; similarly, three libraries were constructed with the RNAs from the spleen erythrocytes of the uninfected control fish. The six libraries were sequenced, and the data were summarized in Table 1. An average 44,186,824 raw reads were obtained, 99.25% of which passed the quality filtering process. After removing ribosomal RNAs, a mean number of 43,858,206 filtered clean reads was obtained from each library, and 82.87%–86.32% of the clean reads were mapped to the flounder genome. A total of 21,398 genes were detected.

### 2.4. Identification of Differentially Expressed Genes (DEGs) Induced by E. tarda

**Figure 3:**
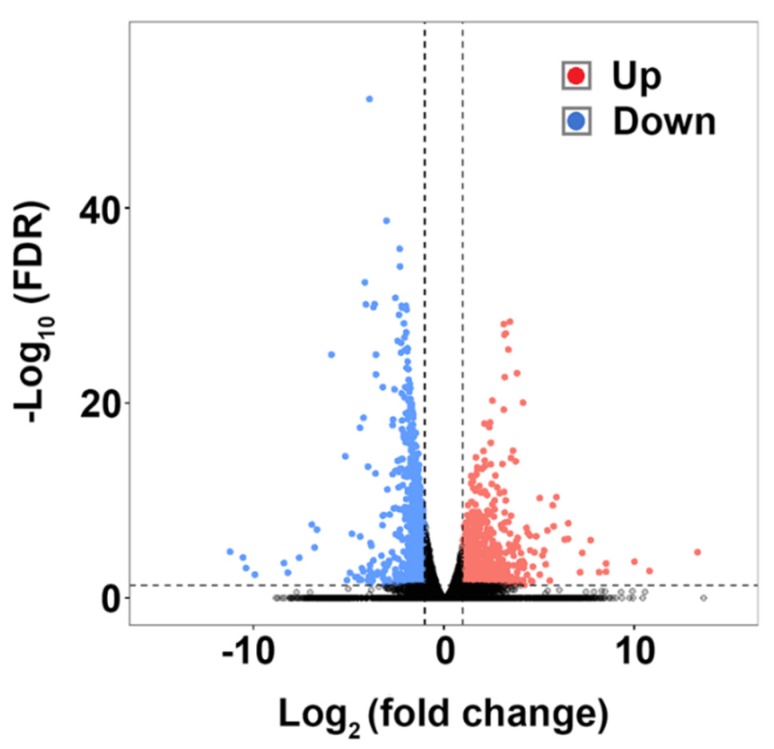
Volcano plot showing the distributions of the differentially expressed genes (DEGs). Each dot represents a gene. Red and blue dots represent up- and downregulated DEGs, respectively.

DEGs were identified by the fold change of the gene expression level (log_2_|FC| > 1) and a false discovery rate (FDR) (FDR < 0.05). Compared to the control group, the *E. tarda*-infected group exhibited 1720 DEGs, 928 and 792 of which were up- and downregulated, respectively. The distributions of the DEGs are shown in Figure 3. To verify the DEGs detected by RNA-seq, 12 DEGs were examined for expression using qRT-PCR. The results showed that the expression trends of the DEGs were in good agreement with that of RNA-seq (Figure S2).

### 2.5. GO and KEGG Enrichment Analysis of the DEGs

**Figure 4:**
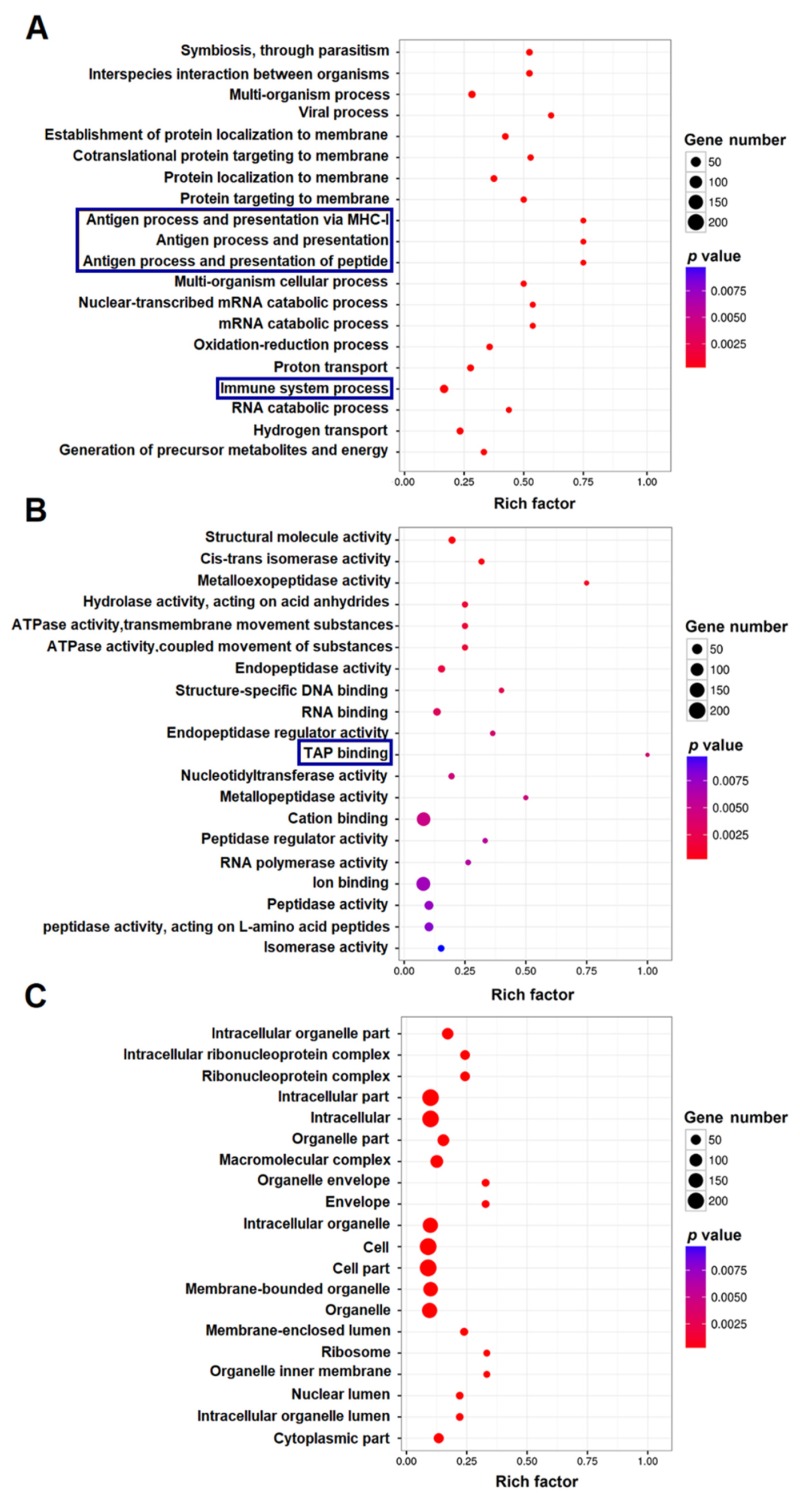
Gene Ontology (GO) enrichment analysis of differentially expressed genes (DEGs). Distribution of the top 20 (level-2) GO terms significantly enriched in the categories of biological process (**A**), molecular function (**B**), and cellular component (**C**) are shown. The immune related genes in (**A**,**B**) are boxed in blue.

GO enrichment analysis indicated that the 1720 DEGs were classified into three main categories: biological process (BP), cellular component (CC), and molecular function (MF). The top 20 significantly enriched GO terms (level-2) in these three categories are shown in Figure 4. Based on the values of rich factor, the top three GO terms in the category of BP were all related to antigen process and presentation, and the other terms associated with immunity were also highly represented in the immune system process (Figure 4A). In the category of MF, the immune related term of transporter associated with antigen processing (TAP) binding ranked top one, while metalloproteinase associated activity (metalloexopeptidase and metallopeptidase activities) ranked top two and three (Figure 4B). In the category of CC, the top three GO terms based on rich factor were ribosome, organelle inner membrane, and organelle envelope (Figure 4C).

To further understand the biological functions of the DEGs, KEGG enrichment analysis was performed. DEGs of 12 immune-related pathways significantly enriched by KEGG analysis are shown in Table 2. These pathways included an intestinal immune network for IgA production, cytokine–cytokine receptor interaction, antigen processing and presentation, spliceosome, hematopoietic cell lineage, C-type lectin receptor signaling pathway, IL-17 signaling pathway, NOD-like receptor signaling pathway, NF-kappa B signaling pathway, Toll-like receptor signaling pathway, cytosolic DNA-sensing pathway, and JAK-STAT signaling pathway. In addition to these immune pathways, the terms of various diseases and infections, including systemic lupus erythematosus, graft-versus-host disease, Parkinson’s disease, Huntington’s disease, Alzheimer’s disease, non-alcoholic fatty liver disease, autoimmune-thyroid disease, measles, influenza A, and Epstein–Barr virus infection, together with allograft rejection and oxidative phosphorylation were also highly represented based on rich factor (Figure S3).

### 2.6. Construction of the Interaction Network Formed by Immune DEGs and Identification of the Hub Genes

**Figure 5:**
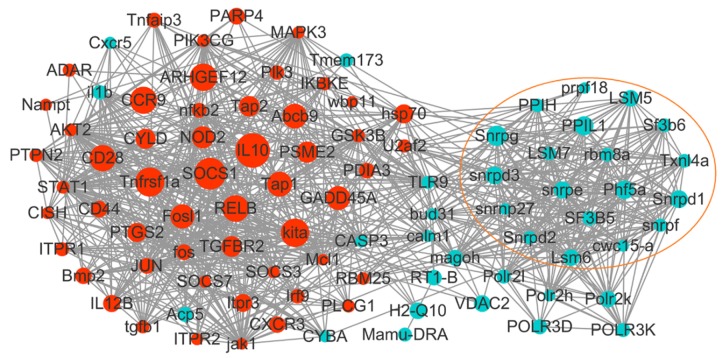
The interaction network formed by immune related DEGs. The nodes in the network represent DEGs, and the lines connecting different nodes denote interactions between the DEGs. Red and blue nodes represent up- and downregulated DEGs, respectively. Node size indicates the log_2_|FC| of the DEGs. The DEGs involved in spliceosome and splicing are enclosed in an orange lined circle.

**Figure 6:**
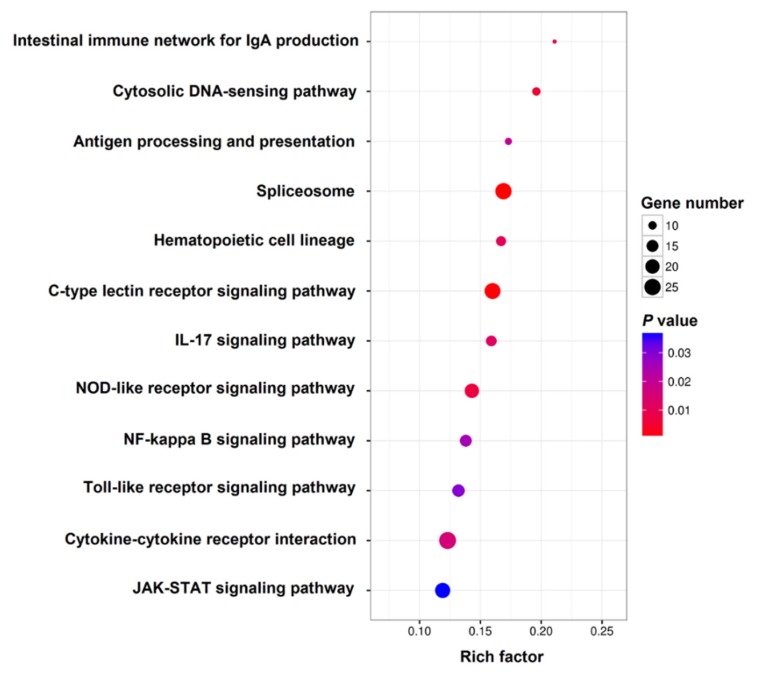
The DEGs that form interactive networks belong to 12 immune related pathways by KEGG analysis. The color and size of the dots indicate *p*-value and DEG number, respectively.

One hundred and eleven DEGs significantly enriched by KEGG analysis were used to construct the immune-related protein interaction network. Ninety-one DEGs (Table S2) turned out to exhibit interactions with each other and form interactive networks (Figure 5). These DEGs were involved in 12 immune related pathways, ranging from pathogen sensing to antigen processing, signal transduction, and effector production (Figure 6). To identify hub genes in the networks, the threshold of log_2_|fold change| of >2 and multiple interaction degree of >3 was further set. As a result, 21 hub DEGs of the networks were identified (Table 3). Most of the hub genes were dramatically upregulated, in particular *IL-10* and suppressor of cytokine signaling 1 (*SOCS1*), which exhibited the highest levels of upregulation (18.6 and 12.6 folds, respectively) and also high levels of interactions with other DEGs (interactive degree of 22 and 29, respectively). Tumor necrosis factor receptor superfamily member 1A, mast/stem cell growth factor receptor kita-like isoform X1, and transcription factor RelB also exhibited a more than 10-fold change in expression and extensive interactions with other genes. Other upregulated hub genes displayed an expressional fold change of between 4.23 to 9.5. Only three hub genes were downregulated, with a fold change of between 4.04 to 4.74 (Table 3).

## 3. Discussion

In this study, we performed both in vitro and in vivo analyses to examine the infectivity of *E. tarda* in flounder RBCs. In vitro infection showed that *E. tarda* was capable of intracellular replication inside RBCs. Consistently, in vivo infection indicated that *E. tarda* was able to invade into the erythrocytes of flounder spleen. These results are in line with the previous reports that showed *E. tarda* is an intracellular pathogen in fish and mammalian models [26,28]. To examine the immune response of RBCs elicited by *E. tarda*, transcriptome analysis was performed, which identified 1720 DEGs in the spleen RBCs of *E. tarda*-infected flounder, indicating a global influence of *E. tarda* on the gene expression of RBCs. It is of note that the top terms in the GO categories of biological process and molecular function were enriched highly with DEGs of antigen process and presentation, and the top terms of the KEGG were enriched predominately with DEGs of immunity, diseases, and infection, indicating an intensive induction of immune defense genes. Ninety-one immune DEGs were found to form high degrees of interactions with each other and constitute a complicated network. Twenty-one hub genes were further identified from the network, which were dramatically regulated by *E. tarda*. The potential functional significances of the key hub genes are discussed below.

### 3.1. Genes Involved in NLRs-Mediated Pathogen Recognition and Downstream NF-κB Activation

Pathogen recognition mediated by PRRs is the first step of host immune response. NLRs are a family of intracellular PRRs that recognize intracellular pathogens and activate downstream signaling events [29,30,31]. *NOD2*, a prototype NLR, has been shown to be an intracellular recognition receptor that can sense the presence of Gram-negative and Gram-positive bacteria in the cytosolic compartment by recognizing muramyl dipeptide on the bacterial cell wall peptidoglycan [32,33,34,35]. In our study, we found that *NOD2* interacted with 15 key immune-related genes and was upregulated by 5.72-fold after *E. tarda* challenge, suggesting that *E. tarda* infection activated the NOD2-mediated NLRs signaling pathway. In mammals, *NOD2* is a positive regulator of NF-κB and, through NF-κB, induces transcription and production of inflammatory mediators [36,37,38,39,40]. In fish, NOD2-induced NF-κB activation has been reported in zebrafish and mrigal [41,42]. In our study, consistent with the upregulated NLRs pathway, NF-κB pathway genes were also significantly upregulated as indicated by the 10.43- and 2.84-fold increase in the expression of *RELB* and *NFKB2* (NF-κB p100 subunit), respectively (Table 3, Table S2). The NF-κB family of transcription factors contain several members, including *RELB* and p100/p52 (*NFKB2*) that form various homo- or heterodimeric complexes and bind to the kappa-B sites in their target genes, leading to activation or repression of the transcription of the target genes. The markedly upregulated expression of *RELB* and *NFKB2* suggests that the NF-κB signaling was active in flounder RBCs during *E. tarda* infection, which was probably at least in part due to the activation of the NLRs pathway. In the interaction networks, *NOD2* displayed interactions with *NFKB2* and other 14 key immune-related genes, which further supported a signaling link between the NLRs pathway and the NF-κB pathway in the context of *E. tarda* infection.

### 3.2. Genes Involved in Antigen Processing and Presentation

Antigen processing and presentation convert pathogenic antigens into immunogenic peptides, which are subsequently exposed on the cell surface and recognized by immunocompetent cells [43,44]. In this study, four hub genes (*TAP1*, *TAP2*, *ABCB9*, and *PSME2*) were significantly enriched in the category of antigen processing and presentation. In mammals, *TAP1* and *TAP2* function to transport antigen peptides into the lumen of the endoplasmic reticulum, where the peptides are loaded onto major histocompatibility complex (MHC) class I molecules [45,46]. *ABCB9* is similar to *TAP1* and *TAP2* and shares high sequence homology with the latter [45]. *PSME2* is known to facilitate antigen process and presentation by strengthening the ability of the 20S proteasome to produce more peptides to bind the MHC-I molecules [47,48]. In our study, *TAP1*, *TAP2*, *ABCB9*, and *PSME2* were all exceedingly upregulated and displayed high levels (degree of 11 and 13) of interactions with other DEGs. These results suggest that flounder RBCs were able to elicit the process of antigen processing and presentation following *E. tarda* challenge.

### 3.3. Genes Involved in Anti-Inflammatory Responses

Inflammation is a protective reaction of the host to clear detrimental stimulations, including pathogens [49]. Anti-inflammatory factors play important roles in the control of inflammatory responses [50,51,52,53,54,55]. These factors include *IL-10* and members of the SOCS family [51,55]. In this study, *IL-10* and *SOCS1* were identified as the top one and top two hub genes that exhibited the highest fold changes (18.63 and 12.61, respectively) in expression upon *E. tarda* infection. *IL-10* is one of the most important anti-inflammatory cytokines in humans and inhibits the expression of TNFα, IL-1, IL-6, IL-8, and other pro-inflammatory cytokines [55,56,57]. *IL-10* also inhibits MHC-II expression and antigen presentation [58,59]. Similarly, in fish, *IL-10* suppresses the expression of pro-inflammatory cytokines in carp, amberjack, mandarin fish, Atlantic cod, and goldfish [59,60,61,62,63]. In mammals, *SOCS1* is induced by a wide range of cytokines and negatively regulates a number of immune signaling pathways [64,65]. In fish, *SOCS1* is known to inhibit the type I/type II IFN signaling pathways in Atlantic salmon and suppress the JAK-STAT signaling pathway in miiuy croaker [66,67]. The dramatically elevated expressions of *IL-10* and *SOCS1* in the RBCs of *E. tarda*-infected flounder suggest inhibition of the inflammatory response in these cells.

In addition to *IL-10* and the *SOCS1*, other DEGs, including *PTGS2*, *TGFBR2*, and *CYLD*, involved in anti-inflammation were also identified among the hub genes in our study. *PTGS2* plays a key role in the generation of prostaglandin E2 (PGE2) that exerts anti-inflammatory function [52,68]. In mammals, PGE2 inhibits the production of pro-inflammatory molecules and enhances the secretion of anti-inflammatory cytokines, such as *IL-10* [69]. *TGFBR2* functions via TGF-β1, which mainly regulates immune suppression/tolerance and anti-inflammatory responses [70,71,72]. *CYLD* is a deubiquitinating enzyme that removes polyubiquitin chains from target proteins such as NF-κB, BCL3, and TRAFs [73,74]. *CYLD* has also been shown to promote anti-inflammation [75,76]. In fish, PGE2 is known to induce *IL-10*, suppress the expression of TNFα and MHC-II, and inhibit the immune response of neutrophils [77,78,79,80,81,82,83,84,85]. TGF-β1 has been reported to block LPS- and TNFα-induced activation of macrophages and peripheral blood lymphocytes in goldfish and grass carp [86,87]. The greatly heightened expressions of *IL-10*, *SOCS1*, *PTGS2*, *TGFBR2*, and *CYLD*, as well as their high degrees of interactions in the networks, observed in our study indicate intense and extensive inductions of anti-inflammatory responses in flounder RBCs following *E. tarda* challenge, which might be the result of bacterial manipulation of the host’s immune reactions to facilitate the invasion and survival of the pathogen in the host.

### 3.4. Genes Involved in Spliceosome and Splicing

In eukaryotic organisms, most genes are firstly expressed as precursor mRNA (pre-mRNA) and then converted to mRNA by splicing [88]. This process is catalyzed by the spliceosome. In our study, 19 key DEGs were significantly enriched in the spliceosome and splicing pathway (Figure 5). Among these genes, *SNRPG*, *PPIL1*, and *PHF5A* were identified as hub genes. In mammals, *SNRPG* is a core component of the spliceosome small nuclear ribonucleoproteins, and *PHF5A* is also a part of the spliceosome and acts as a DNA binding protein [88,89]. *PPIL1* participates in pre-mRNA splicing and is recruited into the spliceosome at the stage of complex B formation [88]. In our study, *SNRPG*, *PPIL1*, and *PHF5A* were all significantly downregulated by 4.04- to 4.74-fold in *E. tarda*-infected RBCs, which is in line with a previous report showing that spliceosome-associated genes were mostly downregulated in rainbow trout RBCs exposed to VHSV [9]. However, in another report of rock bream RBCs exposed to rock bream iridovirus (RBIV), spliceosome-related proteins were mostly upregulated [20]. These results indicate pathogen-specific regulations of spliceosome activity. The strong downregulation in the expression of multiple spliceosome genes observed in our study suggests a systematic inhibition of mRNA processing, which could be a strategy of *E. tarda* to interfere with the expression of the host genes required for pathogen clearance.

## 4. Materials and Methods

### 4.1. Japanese Flounder

Clinically healthy Japanese flounder (average 250 g) were purchased from a local fish farm in Qingdao, China. The fish were acclimatized in the laboratory for one week, during which time the fish were maintained at ~20 °C in aquariums and fed daily with commercial food as reported previously [27,90]. Before the experiment, the fish were verified to be clinically healthy as reported previously [91]. In experiments requiring tissue collection, the fish were euthanized with tricaine methane sulfonate (Sigma, St. Louis, MO, USA) to minimize suffering as described previously [92]. The study with live fish was approved by the Ethics Committee of Institute of Oceanology, Chinese Academy of Sciences (permit No. MB1807) on July 20, 2018.

### 4.2. Isolation of Erythrocytes from the Blood and Spleen of Flounder

Blood was collected from Japanese flounder as reported previously [27]. Briefly, fish were euthanized with tricaine methane sulfonate (Sigma, St. Louis, MO, USA), and the blood was collected from the caudal vein and diluted with PBS (Solarbio, Beijing, China) containing 10 units/mL heparin (Solarbio, Beijing, China). The diluted blood was placed on the top of 1.070 g/mL Percoll (GE Healthcare, Uppsala, Sweden) and centrifuged at 400× *g* for 10 min. Red blood cell pellet was collected and resuspended with RPMI-1640 medium (Gibco, Waltham, MA, USA). The cell suspension was purified with 1.070 g/mL Percoll for 2–3 times to obtain high purity RBCs. The purified RBCs were added into RPMI-1640 medium containing 10% calf serum (Gibco, Carlsbad, CA, USA), 100 U/mL penicillin (Solarbio, Beijing, China), 100 µg/mL streptomycin (Solarbio, Beijing, China), and 50 µg/mL gentamicin (Solarbio, Beijing, China). To isolate spleen RBCs, spleen tissues were collected from the fish aseptically and gently ground in a cell culture dish (Corning, Jiangsu, China) containing 10 units/mL heparin (Solarbio, Beijing, China). RBCs were then isolated and purified as described above.

### 4.3. In Vitro Infection of E. tarda in RBCs

Examination of the intracellular replication of *E. tarda* was carried out as reported previously [93]. Briefly, RBCs were incubated with *E. tarda* as described above at a MOI of 10. After 3 h of incubation, the RBCs were collected by centrifugation at 4 °C and gently washed twice with PBS. The bacteria attached to the surface of RBCs were killed by adding gentamicin (200 µg/mL) and incubation at 20 °C for 2 h. The cells were washed two times with PBS and cultured in RPMI-1640 medium containing 10 µg/mL gentamicin for 0 h, 2 h, or 4 h. At each time point, 1% Triton X-100 was added to lyse the RBCs, and the lysate was diluted and plated onto LB agar plates. After overnight incubation at 28 °C, the colonies that appeared on the plates were counted. The experiment was performed three times.

### 4.4. In Vivo Infection of E. tarda in Flounder

*E. tarda* was suspended in PBS to a final concentration of 5 × 10^8^ colony forming units (CFU) mL^−1^. Flounder were randomly divided into two groups. The experimental group was intraperitoneally injected with 200 μL of the above bacterial suspension per fish, and the control group was injected with the same volume of PBS. At 12 h and 24 h post-infection (hpi), blood and spleen tissues were taken aseptically from the fish (3 individuals at each time point from each group) and isolated and purified as described above. The purified RBCs were divided into two parts and used to determine the numbers of cell surface-attached *E. tarda* and intracellular *E. tarda*. For this purpose, one part of the PBCs was directly lysed, while the other part was firstly incubated with gentamicin (200 µg/mL) for 2 h to kill extracellular *E. tarda* and then lysed. The cell lysates from the two parts were each plated onto LB agar plates, which were incubated as described above, and the colony numbers were counted. The number of cell surface-attached *E. tarda* was obtained by subtracting the bacterial number in the second part of lysate from the bacterial number in the first part of lysate.

### 4.5. RNA Sequencing Library Construction and Sequencing

Flounder were divided into two groups and infected with *E. tarda* or treated with PBS (control group) as described above. At 24 hpi, RBCs were collected from the spleen of the fish (6 fish from each group) and purified as described above. The purified RBCs were inspected by light microscopy to confirm purity (Figure S1). For transcriptome analysis, six RNA-seq libraries (triplicates of the infected group (2 fish/group) and the uninfected control group (2 fish/group)) were constructed using the purified RBCs. Total RNA extraction was performed using Trizol RNA extraction reagent (Invitrogen, CA, USA) following the manufacturer’s protocol. The quality of the purified RNA was assessed using Agilent 2100 Bioanalyzer. RNA integrity was determined by agarose gel electrophoresis. The sequencing libraries were constructed according to the Illumina’s standard protocol as previously reported [94]. Briefly, mRNA was enriched from total RNA using Oligo (dT) beads (Qiagen, Hilden, Germany) and fragmented into short fragments, which were reverse transcribed into first-strand cDNA. Second-strand cDNA was then synthesized with DNA polymerase I (Thermo Scientific, Waltham, MA, USA), dNTP, and RNase H. The cDNA fragments were purified, end repaired and added with poly (A), and ligated to Illumina sequencing adapters. Finally, the libraries were sequenced with the Illumina Hiseq-novaseq 6000 platform by the Gene Denovo Biotechnology Co. (Guangzhou, China).

### 4.6. Data Preprocessing

The raw reads of the libraries were filtered by removing low-quality reads, including reads containing nucleotide with a *Q* quality score of ≤20, reads containing adapters, and reads with undetermined nucleotides larger than 10%, before mapping to the ribosome RNA (rRNA) database in Bowtie 2 (v2.2.8) [95]. The rRNA-mapped reads were removed, and the remaining reads were mapped to the Japanese flounder genome sequence (GenBank project accession PRJNA369269) with TopHat2 (v2.0.3.12) [96]. The reconstruction of the transcripts was conducted with Cufflinks (v2.2.1) [97]. The abundance of each transcript was quantified with the software RSEM v1.2.19 [98]. The gene expression level was normalized by using the FPKM (Fragments Per Kilobase of transcript per Million mapped reads) method [99].

### 4.7. Identification, Validation, and Functional Enrichment Analysis of Differentially Expressed Genes (DEGs)

Differential expression analysis was carried out using the R package edgeR (v3.12.1) (http://www.r-project.org/). The exact negative binomial test [100] was applied to perform pairwise comparison between the infected group and the control group. Transcripts with a false discovery rate (FDR) of <0.05 and an expression fold change (FC) of >2 (log_2_|FC| > 1) were considered as DEGs. The expressions of 12 DEGs were validated by quantitative real-time reverse transcription-PCR (qRT-PCR) as reported previously [26,101]. Briefly, gene-specific primers were designed using Primer designer of NCBI (https://www.ncbi.nlm.nih.gov) and listed in Table S1. qRT-PCR was carried out in a QuantStudio 3 Real-Time PCR Systems (Thermo Fisher Scientific, CA, USA) using SYBR Premix Ex Taq II (Takara, Dalian, China) following the manufacturer’s protocol. The melting curve analysis was conducted to confirm that the specific PCR product was amplified and detected. The expression levels of the target genes were analyzed using the comparative threshold cycle method (2−ΔΔCT) with β-actin as an internal reference [2]. The DEGs were subjected to Gene Ontology (GO) functional analysis and Kyoto Encyclopedia of Genes and Genomes (KEGG) pathway enrichment based on GO database (http://geneontology.org) and KEGG database (http://www.genome.jp/kegg/). A *p*-value of <0.05 was set as a threshold to identify significantly enriched GO terms and KEGG pathways using hypergeometric test.

### 4.8. Construction of Interaction Network and Hub Genes Identification

The immune-related DEGs in Table 2 were used to construct protein–protein interaction (PPI) networks using the String software (http://string-db.org/) [102] as reported previously [26,101]. The hub genes were further identified from PPI networks based on the following degrees: log_2_|FC| > 2 and PPI > 3.

### 4.9. Statistical Analysis

All experiments were performed three times or in triplicate, and graphic representation and statistical analyses were carried out with Graphpad Prism 6 (www.graphpad.com). Data were analyzed with the Student’s *t*-test, and statistical significance was defined as *p* < 0.05.

## 5. Conclusions

In this study, transcriptome analysis reveals a large scale immune response induced by *E. tarda* in flounder RBCs. A core set of DEGs significantly enriched to 12 immune-related pathways were found to form complicated interaction networks. The hub genes of the networks are involved in key immune processes that, for some, promote pathogen elimination and, for some others, may facilitate pathogen infection. These results indicate that flounder RBCs are capable of mounting profound immune responses in an effort to fight against *E. tarda*, but some of the immune responses are likely manipulated by *E. tarda* to the advantage of optimum bacterial invasion. Our results add new insights into the immune role of flounder RBCs in association with bacterial infection and provide a genetic basis for future study of the immune mechanisms of flounder RBCs. In future studies, it should be interesting to compare the immune responses of RBCs in different tissues and see whether there is any tissue-specificity in the response.

## Figures and Tables

**Table 1:** Quality of RNA sequencing. C, control group; E, *E. tarda*-infected group. Each group was triplicated and shown as -1, -2, and -3.

Samples	Read Length (bp)	Raw Reads	Clean Reads	Clean Read Ratio (%)	Mapped Read Ratio (%)
C-1	150	52,745,248	52,367,850	99.28	86.32
C-2	150	39,343,878	39,031,230	99.21	85.97
C-3	150	52,836,746	52,477,326	99.32	85.25
E-1	150	38,019,958	37,735,932	99.25	85.49
E-2	150	40,950,324	40,635,040	99.23	84.35
E-3	150	41,224,792	40,901,860	99.22	82.87

**Table 2:** Summary of the 12 significantly enriched KEGG pathways related to immunity.

Pathway	DEG Number	Rich Factor	*p*-Value
Intestinal immune network for IgA production	8	0.211	0.008804
Cytosolic DNA-sensing pathway	10	0.196	0.006066
Antigen processing and presentation	9	0.173	0.020065
Spliceosome	25	0.169	0.000232
Hematopoietic cell lineage	12	0.167	0.010609
C-type lectin receptor signaling pathway	24	0.160	0.000698
IL-17 signaling pathway	13	0.159	0.012083
NOD-like receptor signaling pathway	20	0.143	0.007153
NF-kappa B signaling pathway	15	0.138	0.025076
Toll-like receptor signaling pathway	16	0.132	0.029570
Cytokine–cytokine receptor interaction	27	0.123	0.015126
JAK-STAT signaling pathway	22	0.119	0.036321

**Table 3:** Summary of the 21 hub DEGs. The “+” and “–” symbols before the fold change number indicate up- and downregulation, respectively.

Gene Name	Description	Interactive Degree	Fold Change
*IL10*	Interleukin-10	22	+18.63
*SOCS1*	Suppressor of cytokine signaling 1	29	+12.61
*TNFASF1A*	Tumor necrosis factor receptor superfamily member 1A	16	+11.19
*KITA*	Mast/stem cell growth factor receptor kita-like isoform X1	33	+10.44
*RELB*	Transcription factor RelB homolog isoform X1	40	+10.43
*ARHGEF12*	Rho guanine nucleotide exchange factor 12	7	+9.50
*CD28*	T cell specific surface glycoprotein CD28	14	+8.58
*TAP1*	Antigen peptide transporter 1	13	+7.42
*ABCB9*	ATP-binding cassette subfamily B member 9	13	+7.34
*FOSL1*	Fos-related antigen 1	21	+6.96
*GADD45A*	Growth arrest and DNA damage protein GADD45 alpha-like	11	+6.72
*NOD2*	Nucleotide-binding oligomerization domain containing 2	15	+5.72
*PSME2*	Proteasome activator complex subunit 2	11	+5.23
*TGFBR2*	TGF-beta receptor type 2	10	+4.96
*TAP2*	Antigen peptide transporter 2	11	+4.93
*SNRPG*	Small nuclear ribonucleoprotein polypeptide G	25	−4.74
*CYLD*	Ubiquitin carboxyl-terminal hydrolase CYLD-like	7	+4.69
*PTGS2*	Prostaglandin G/H synthase 2	16	+4.27
*PPIL1*	Peptidyl-prolyl cis-trans isomerase-like 1	20	−4.27
*HSP70*	Heat shock 70 KDA protein	28	+4.23
*PHF5A*	PHD finger-like domain-containing protein 5A	23	−4.04

## Supplementary Materials

The following are available online at https://www.mdpi.com/1422-0067/21/9/3094/s1, Table S1. List of the primers used for qRT-PCR. Table S2. List of the 91 immune-related DEGs used in Figure 6. Figure S1. Microscopic observation of purified flounder spleen red blood cells (RBCs). Figure S2. Comparison of the relative expressions of selected DEGs by qRT-PCR and RNA-Seq. The relative expression levels of 12 DEGs were determined by qRT-PCR, and the results are compared with that of RNA-Seq. Red dotted line represents the control group. Figure S3. The top 20 enriched KEGG pathways of differentially expressed genes (DEGs). The color and size of the dots indicate *p*-value and DEG number, respectively.
