# Supplementary material for: Transcriptome Analysis of Paralichthys olivaceus Erythrocytes Reveals Profound Immune Responses Induced by Edwardsiella tarda Infection
Source: Int J Mol Sci. 2020 Apr 28;21(9):3094. doi: 10.3390/ijms21093094 (PMC7247156; doi:10.3390/ijms21093094)
Supplement: Supplementary file 1 [file ijms-21-03094-s001.zip › Table S1.docx]

**Supplementary data**

**Table S1.** List of the primers used for qRT-PCR.

| **Gene name** | **Gene ID** | **Forward primer (5′-3′)** | **Reverse primer (5′-3′)** |
| --- | --- | --- | --- |
| JUN | XM_020081378.1 | GCGGAGAGAGCAAAGACTGT | TGGATCACGGCCACTGTTAC |
| FOS  RELB  SOCS1  TNFASF1A  LSM5  LSM7  SNRPE  SNRPG  SNRPD3  SF3B5 | XM_020113934.1  XM_020110583.1 XM_020110805.1  XM_020093063.1 XM_020109691.1 XM_020080405.1 XM_020086360.1  XM_020088938.1  XM_020081573.1  XM_020087157.1 | CTCTGCAAGCTGAAACGGATGT  GCCTGTCAGACTTCGAGCTCAT  CTGTCCTACCACGCCAAGAG  GGTCGCTCTCATCACTCACC  GTATCGGCTCCCGGATTCAC  CTTCCACTGTGCAGGATAAAGA  CCGCTGGTATTTTTGTGGCG  GCCGGAAGTGTGCTGTTTAG  TGCATTGTGGGTAAGCGGAA  TTATCGAAGAGTCTGTGATTCCCG | GATGGAGACCACTGGGAAGT  CTAGCACCGGCTGTGACG  CGCTTCCTGCACAATTCCTG  ACAGGATCAACTCGCAAGGG  GGTGTGATTTCAAACTCCGTCA  CGTCTGGATCACGCATGTACT  CCTGGATCCGTGAGCGATTT  CCATGAACTTCTTCAGCTCGGG  TTCAACTGTCTGGACTCTGGTG  GAGCAGGTCGAAATGTCCCA |
| CYLD  β-actin | XM_020107346.1  XM_020109620.1 | GAGTCAGACCTTTGGTCCCC  TCAGCAAGCAGGAGTACGATG | TCCATGTCTGTGGAGATCCGA  GAGCTGAAGTTGTTGGGCGT |


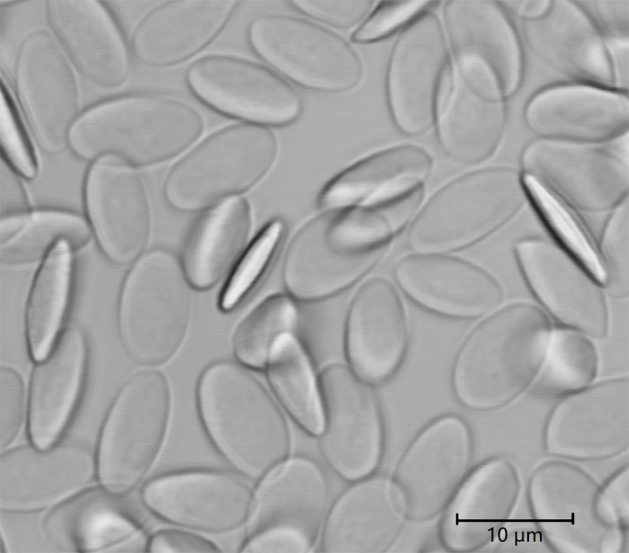


**Figure S1.** Microscopic observation of purified flounder spleen red blood cells (RBCs).


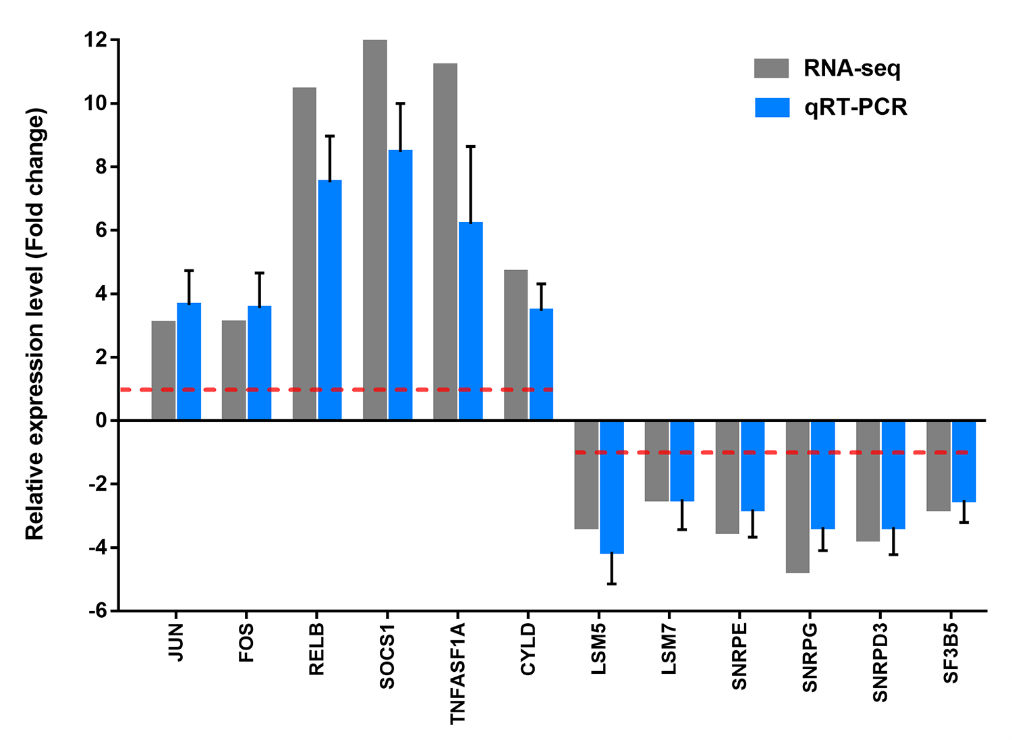


**Figure S2**. Comparison of the relative expressions of selected DEGs by qRT-PCR and RNA-Seq. The relative expression levels of 12 DEGs were determined by qRT-PCR, and the results are compared with that of RNA-Seq. Red dotted line represents the control group.


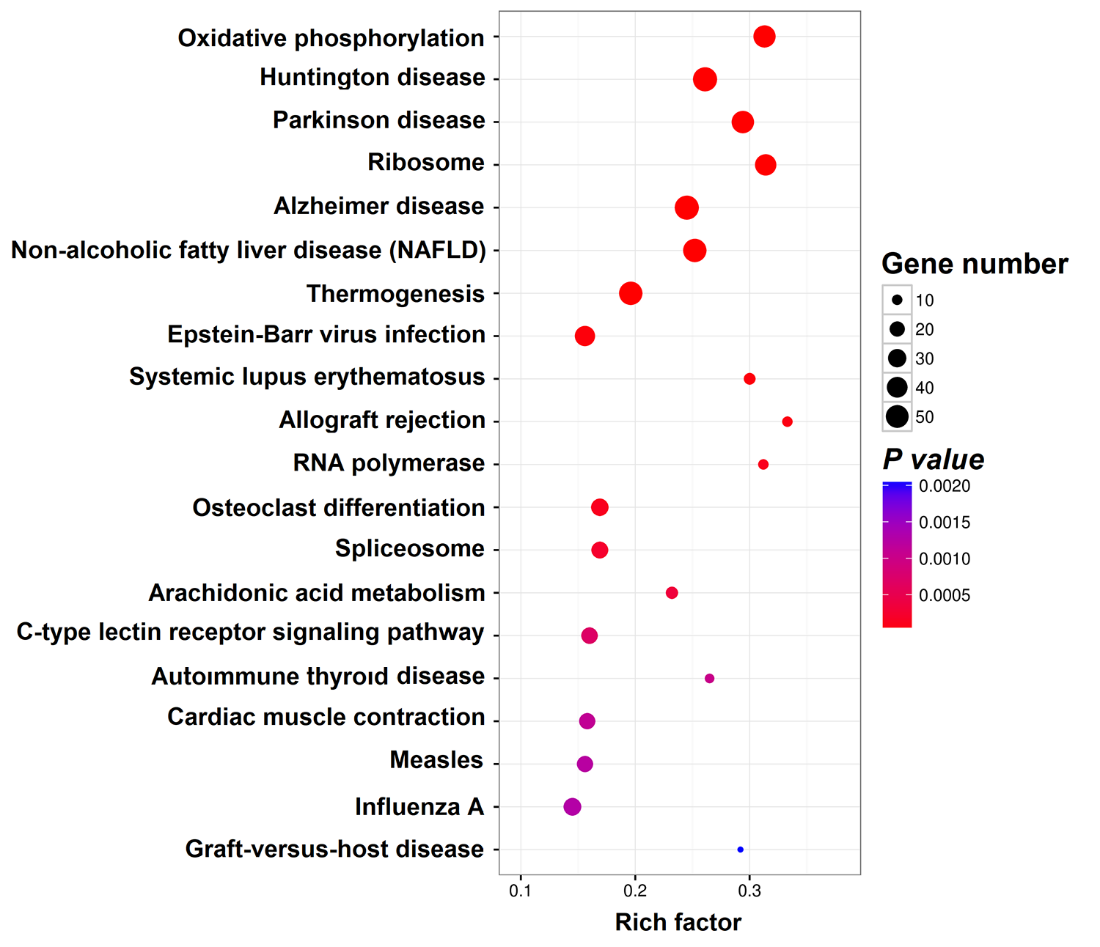


**Figure S3**. The top 20 enriched KEGG pathways of differentially expressed genes (DEGs). The color and size of the dots indicate p-value and DEG number, respectively.
